# Death and Railroads

**Published:** 1856-03

**Authors:** 


Death and Railroads.—Of all modes of travel, that on railroads is safest for life and limb, if the official statement be true, that of twelve million of passengers transported over the main railroads of New York during 1855, only twelve were killed, and of these, eleven were standing on the platform. The safest place on a rail car is the inside, about the centre of the car, if there be no stove there, and on the end of the seat, next to the aisle.

